# Physical Activity Programs with Post-Intervention Follow-Up in Children: A Comprehensive Review According to Categories of Intervention

**DOI:** 10.3390/ijerph13070664

**Published:** 2016-06-30

**Authors:** Sally Nguyen, Anna-Luisa Häcker, Melanie Henderson, Tracie Barnett, Marie-Eve Mathieu, Linda Pagani, Jean-Luc Bigras

**Affiliations:** 1 Department of Pediatrics, CIRCUIT Center, University of Montreal, Montreal, QC H3T 1J4, Canada; sally.nguyen7@gmail.com (S.N.); melanie.henderson.hsj@gmail.com (M.H.); 2 Technische Universität München, Munich 80992, Germany; anna.haecker@tum.de; 3 Sainte-Justine UHC Research Center, University of Montreal, Montreal, QC H3T 1C5, Canada; tracie.barnett@gmail.com (T.B.); me.mathieu@umontreal.ca (M.-E.M.); Linda.s.pagani@umontreal.ca (L.P.); 4 Epidemiology and Biostatistics Unit, INRS-Institute Armand-Frappier, Laval, QC H7V 1B7, Canada; 5 Department of Kinesiology, University of Montreal, Montreal, QC H3T 1J4, Canada

**Keywords:** physical activity, cardiovascular risk factors, children, adolescents, intervention

## Abstract

Only 9% of Canadian children meet the National Guidelines of 60 min of daily moderate-to-vigorous intensity physical activity. The aim of this review is to assess the mid- and long-term effectiveness of physical activity interventions and their impact on cardiovascular risk factors in children. We assessed the success of interventions within three different categories: those using a behavioural and social approach, an informational approach or an environmental approach. The average number of children included in these studies was 860 (range of 30–5106); the age range was from 2 to 18 years; and the mean intervention duration was 1607 min (range of 12–8160 min). The length of follow-up post-intervention averaged 13 months (ranging from 0.25 to 96 months). A positive impact on physical activity was found in 74% and on any measured outcomes in 90% of the studies reviewed. However, the benefits of physical activity interventions decreased with longer follow-up. Regardless of the approaches, physical activity interventions improved cardiovascular risk factors. However, the challenge of any program is to maintain beneficial effects once the intervention is completed. These findings will inform the development of future intervention programs in order to optimize sustained cardiovascular benefits.

## 1. Introduction

Physical activity has been proven to entail health benefits for children and adolescents. Even a minimal amount of physical activity could be favorable for those at high risk of cardiovascular disease and could decrease the risk of weight-related chronic diseases, such as obesity, hyperinsulinemia, impaired glucose tolerance, dyslipidemia and hypertension [1,2]. These chronic diseases, once considered to be adult conditions, are increasing at a surprising rate in pediatric populations. Among the most prevalent cardiovascular risk factors in children is obesity. This condition also causes social discrimination among children, emotional problems, school problems and functional restrictions [3]. Excessive weight during childhood and adolescence persists into adulthood. A high body mass index in childhood and adolescence is also associated with higher morbidity and mortality rates in adulthood [4].

Despite the compelling evidence of the benefits of physical activity on health, only 9% of Canadian children between five and 17 years meet the daily recommendations of international guidelines of 60 min of moderate-to-vigorous intensity physical activity [5,6,7]. Sedentary behaviours, such as watching television, surfing the Internet and playing video games, have increased in popularity over the past few years and have contributed to children’s low physical activity levels and obesity [8]. Unhealthy eating habits have also played a major role in the growing epidemic of childhood obesity, with today’s easy access to low-nutrient and energy-dense foods [4]. For all those reasons, primary prevention for cardiovascular diseases and childhood obesity should begin as early as in childhood [4,8]. Many prevention programs have been proven efficient in preventing the emergence of cardiovascular risk factors [9,10,11]. Some comprehensive reviews of those programs have also illustrated their effectiveness in lowering cardiovascular risk factors and, in particular, increasing physical activity [1,12,13]. Physical activity is an established risk factor for cardiovascular disease in youth and is the focus of this review. The aim of this review was to further analyze whether the success of a program comes from an approach that addresses the child’s behaviour and his or her family, the knowledge of a healthier lifestyle or the environment. This categorization was already used in a review including a preschool population [13]. Our comprehensive review will focus on school-age children.

## 2. Methods

Program interventions in children that aimed to increase physical activity while improving other cardiovascular risk factors were reviewed. The intervention programs were either focused exclusively on physical activity or included components such as decreased sedentary behavior, healthier eating habits or community involvement. As the etiology of being overweight and obesity is multifactorial, the interventions often include multiple approaches. To facilitate the implementation of a healthy lifestyle for children, various factors in a child’s daily life can be influenced. These factors include the child’s behavior and his or her family, as well as knowledge about physical activity and the environment. Therefore, these studies were categorized as either a behavioral or a social approach, an informational approach or an environmental approach. This categorization was inspired by the systematic review by Kahn et al. [13].

Behavioral and social approaches aim at teaching skills that are necessary for a successful initiation and maintenance of behavior changes to increase physical activity. Therefore, it is important to learn how to create an environment that facilitates, supports and enhances behavioral changes. One part of the interventions may contain individual and group counseling, which can include peers and families, while the other part may consist of interactive physical activity, such as training programs.

Informational approaches aim to increase physical activity by increasing knowledge, improving attitudes about the benefits of physical activity and increasing awareness of opportunities for physical activity within a community. This method provides necessary information to empower and motivate people to change their behavior, with the ultimate goal to maintain that beneficial change over time. The main focus of this approach is knowledge and cognitive skills, but it can also focus on methods and information to overcome the possible barriers to a successful change in behavior.

Environmental and policy approaches aim to change the structure of physical and organizational environments to provide safe, attractive and convenient places for physical activity to support people in enhancing their physical activity level and adopting healthier behaviors. The main focus of this approach is less on the individual and more on the community and the environment surrounding the chosen community.

Inclusion criteria for studies in this review were: (1) publication in English language; (2) publication from January 1990 to July 2012 inclusively; (3) interventions targeting children in the 0–18 years of age range; (4) interventions with a physical activity component aiming to reduce obesity in children and including an objective assessment of obesity; (5) published results of the intervention with at least one follow-up period post-intervention. Exclusion criteria for studies in this review were: (1) review articles; (2) articles published without results or with pre-intervention data results only; (3) articles with only an abstract available. The search for relevant papers was conducted by a librarian in Medline in July 2012 with the keywords: “effective” AND “physical activity” OR “exercise” AND “intervention” OR “program” AND “cardiovascular prevention” AND “children”. Five hundred and fifty nine potential eligible abstracts were identified from this search strategy. After the duplicated citations were removed, abstracts were reviewed by a single reviewer to determine whether or not the article was pertinent for this review. Full-text articles were then obtained and saved in EndNote. Three hundred and fifty two articles were rejected after screening titles and abstracts, and 183 full-text articles were retrieved from the Medline search. We applied our inclusion/exclusion criteria on those 183 remaining articles and retained 50 articles for our review, representing 47 different interventions.

Data collection retrieved from each study included information on: the study design and type of intervention; study objectives; population; sample size and characteristics; number and percentage of drop-outs; intervention duration and frequency of intervention; duration of follow-up post-intervention; outcomes and results. We determined that a study had a positive impact when the authors reported significant differences over time pre-/post-intervention. All studies established statistical significance on *p* ≤ 0.05.

## 3. Results

After applying the inclusion criteria, a total of 50 articles were retained for this review. Among those articles, three reported the long-term outcomes of an initial intervention. Thirty-one of those studies included a control group (Table 1), while 19 studies did not (Table 2). The majority of studies were conducted in the USA (*n* = 23), with others in Europe (*n* = 15), as well as in Australia (*n* = 5).

The average sample size was 860 (range of 30–5106) children. The age range was from 2 to 18 years old. The average drop-out rate was 24% (range of 0–76%). The average duration of follow-up was 12.1 months (range of 0.25–96 months). Behavioral intervention programs had an average duration follow-up of 9.7 months, informational of 10.4 months and environmental intervention programs of 36.0 months. The average intervention duration (i.e., the time during which the intervention content was being delivered) of all programs was 1607 min (range of 12–8160 min). The behavioral approaches had an average intervention duration of 1704 min and the informational approaches 390 min, and there was no data available for the environmental approaches. Physical activity was measured by self-report in 54% of the studies, including questionnaires and interviews [14,15,16,17,18,19,20,21,22,23,24,25,26,27,28,29,30,31,32,33,34,35,36,37,38,39,40]. Thirty-two percent of the intervention programs measured objectively the outcomes, including the use of accelerometers and pedometers to track daily activity and treadmills, shuttle-runs and one-mile-runs to measure the fitness level [41,42,43,44,45,46,47,48,49,50,51,52,53,54,55,56]. Accelerometry was used in seven (14%) studies [41,42,46,48,51,55,56]. Among those studies, five were successful in improving physical activity [41,48,51,55,56]. Three studies reported an improvement in the minutes of moderate-to-vigorous intensity by 7% [56], 11% [48] and 16% [41]. Two studies reported an improvement in counts by 7% [51] and 14% [55]. Two studies had no significant improvement in physical activity post-intervention [42,46]. Pedometry was used in four (8%) studies [43,44,45,49]. Among those studies, three were successful in improving physical activity. Their results showed an improvement of 9% [44], 23% [43] and 71% [49]. One intervention did not improve physical activity as measured by pedometers [45].

**Figure 1:**
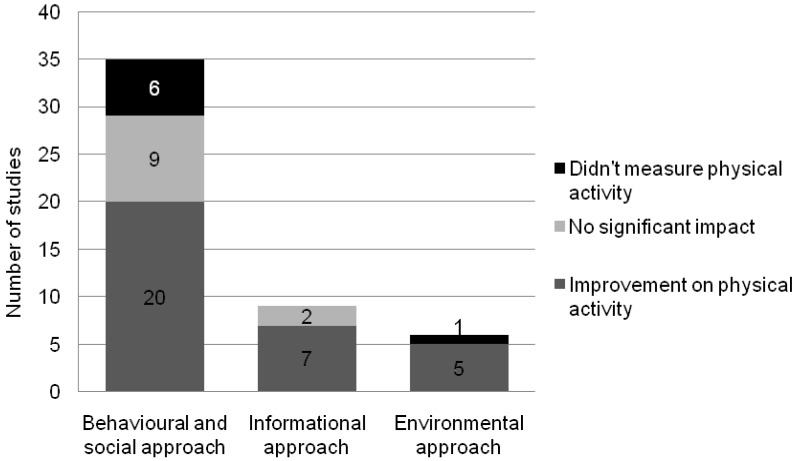
Impact of interventions on physical activity.

In total, 32 (74%) studies had a positive impact on physical activity during, immediately after or at follow-up of the intervention [14,15,16,18,20,21,22,23,24,25,28,29,30,31,34,35,36,37,38,39,40,41,43,44,47,48,49,50,51,54,55,56,57]. Eleven interventions (24%) did not find any statistically-significant effects on physical activity at any time point [17,19,26,27,32,33,42,45,46,52,53]. Figure 1 shows that among seven different interventions that aimed to increase physical activity in order to improve cardiovascular outcomes, none reported results on physical activity [57,58,59,60,61,62,63]. Although these studies did not report data on physical activity, they did report the impact of the intervention on anthropometric changes, blood pressure, cholesterol, glycemia, insulin and knowledge.

**Figure 2:**
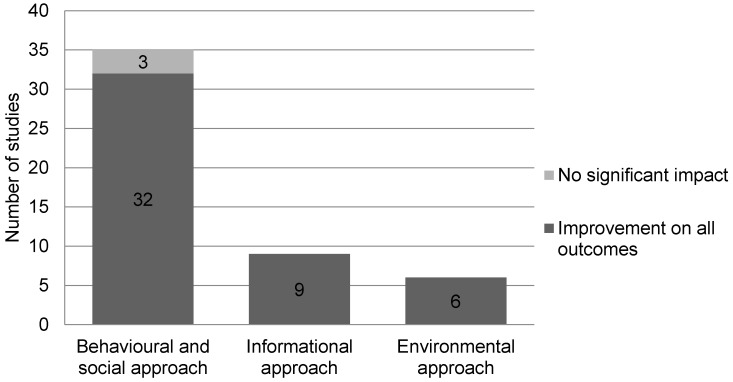
Impact of interventions on all outcomes.

Furthermore, we assessed positive impacts on measured cardiovascular disease risk factors, such as anthropometry, sedentary behaviour, nutritional habits, psychological and other effects in the studies where possible. Figure 2 shows that positive impacts were noted in 45 (90%) of the studies having measured these outcomes [14,15,16,18,19,20,21,22,23,24,25,26,28,29,30,31,32,33,34,35,36,37,38,39,40,41,42,43,44,47,48,49,50,51,52,53,54,55,56,57,58,59,60,61,62]. Among these studies, 13 had a beneficial impact on anthropometry [24,25,28,35,47,50,52,55,57,58,59,60,63], and eight studies reported a decrease in sedentary behavior [19,24,25,28,30,32,39,56]. Nutritional habits were improved in 15 studies [18,20,21,25,29,30,35,39,42,45,47,53,55,62,63], and positive changes in psychological well-being were reported in eight studies [14,18,23,24,32,34,59,62]. Psycho-social support or parental support had a favorable impact on the outcomes [24,48,52]. Since only a few studies provided information on parental support/psycho-social support and given that these were not the primary outcomes of interest, we felt that including these outcomes would be including an excessive amount of information in our table, making it harder to interpret. Twenty-two studies reported positive impacts on other cardiovascular risk factors, such as knowledge about a healthier lifestyle, cardiovascular fitness, blood pressure and alcohol, drug and cigarette use [14,15,18,22,24,26,28,33,35,37,39,47,50,51,52,53,59,60,61,62,63].

In order to understand the nature of the various interventions, the studies were classified into the above mentioned three categories of interventions. There were 35 studies that had a behavioral and social approach; nine had an informational approach; and six had an environmental approach. If a study could be assigned to two or three classifications, it got classified as part of the dominating approach cited by the authors of the article.

In the behavioral and social approach, 20 (67%) interventions reported significant improvements on physical activity at any-point-in-time [14,15,16,18,20,21,22,23,24,25,28,29,41,43,44,47,48,49,50,51]. Horne et al. [43] reported a sex-related difference in the effect of the intervention on physical activity over time. Both boys and girls had increased physical activity during the intervention, but the effect was only maintained for girls, three months later. Furthermore, four interventions measured stronger effects on physical activity at follow-up compared to immediately after the intervention [16,20,22,44]. The intervention program proposed by Jemmott et al. [18] measured the highest improvement of physical activity at the end of the intervention program. While the benefits still existed at the second and third follow-up, they were somewhat attenuated [18]. Several studies reported that the beneficial effect of the intervention studied was lost with time [15,21,23,25,28,48].

Within the informational approach, seven (78%) interventions reported improvements on physical activity [30,31,34,35,36,37,54]. Manios et al. [54] analyzed boys and girls separately and reported a significant effect on physical activity after the intervention in both sexes [37]. The improvement, however, was only maintained in boys at four years’ follow-up [54].

Five (83%) interventions using the environmental approach reported improvements on physical activity at follow-up [38,39,40,55,56]. The intervention program reported by Webber et al. [56] did not show any improvement in physical activity immediately at the end of the intervention; however, physical activity was significantly increased 12 months later in 3378 children.

## 4. Discussion

The aim of this review was to analyze whether the success of a program comes from a design that addresses the child’s behaviour and his or her family, the knowledge of a healthier lifestyle or the environment.

The three different designs of intervention programs showed different results in improving physical activity. Behavioral approaches reported success in improving physical activity in 20 interventions (67%). Informational approaches reported success in improving physical activity in seven interventions (78%). Environmental approaches reported success in improving physical activity in five interventions (83%). When looking at the efficiency and the reproducibility of an intervention, it is very useful to have objective measurements of physical activity. The fractions of programs within the behavioral and environmental approaches relying on objective measurements of physical activity were similar (43% and 40%, respectively). Meanwhile, only 14% of the informational approaches used an objective measure. A possible reason that behavioral and environmental approaches used more objective measurements could be that these intervention programs entail higher costs, and an evaluation of the efficiency of the programs becomes mandatory. The design of informational approaches varies and sometimes does not require significant amounts of resources. For example, Chen et al. [35] was successful in improving anthropometric measurements and physical activity by mailing information to families. However, the benefits of the program were self-reported and not measured objectively.

The durations of intervention and follow-up are associated with different success rates. Although the behavioural and informational programs had similar success, the behavioural programs spent more time with the children. No studies reported the frequency or the duration of the intervention in the environmental programs. The time reported in Table 1 and Table 2 for environmental programs represents the global time during which the children were exposed to the specific environment. Many authors outlined the importance of time length to obtain good outcomes. The program intervention of Jemmott et al. [18] conducted a cognitive-behavioral health-promotion intervention and achieved a positive effect on physical activity, nutritional habits, psychological effects and knowledge, but mentioned having even stronger effects by stretching the intervention program over a longer period of time. Araujo-Soares et al. [22] had increasing beneficial effects on physical activity over time through their intervention based on social cognitive theory, self-regulation theory and planning. They explained these outcomes by the importance of self-monitoring and coping planning in behavior changes. Other studies, such as De Bourdeaudhuij et al. [16], emphasize the importance of the duration of follow-up by reporting even stronger effects after a longer time of follow-up and with only two assessments during this time frame. One environmental approach by Webber et al. [56] did not show any significant improvements on physical activity at the end of the intervention, but reported an improvement 12 months later. This intervention included operant learning theory, social cognitive theory, organizational change theory and the innovation model in a social-ecologic setting. The authors repeated an assessment one year later and showed a modestly significant increase of 1.6 min of moderate-to-vigorous physical activity per day, which could indicate that a broader, longer intervention with more resources is needed.

Mean drop-out rates were similar among studies in the behavioral (22%, range of 0%–73%), informational (29%, range of 23%–41%) and environmental (26%, range of 21%–34%) approaches. It was not possible from the available data to understand the causes for the wide dispersion of drop-out rates. We considered reasons for drop-out in physical activity interventions with control groups having the highest drop-out rates (≥30%), which included: lack of time or personnel power [26], logistical factors, such as lack of computers or funding [24], as well as children changing schools [22,37] or classrooms that chose other priorities than physical activity [17]. Finally, other factors, such as older age, being a male student or having less sitting time for explanations to learn about the intervention, had a negative impact on the drop-out rates [16,17].

Comparisons of study results remains difficult, given the heterogeneity across types of interventions, methodology and target populations. No one category of intervention emerged as more successful than another; however, we were able to identify some important factors in the design of interventions that led to a positive impact on the outcomes of the studies. We identified that psycho-social support, including parental support, was an important factor for the success of an intervention program regardless of the design. The randomized controlled trial by Sacher et al. [24] found very good improvements on all outcomes that were measured. The intervention consisted of a multicomponent healthy lifestyle program that was provided to the families through education, skills training and motivational enhancement. The program required the participation of the children, as well as one parent. The effect was sustained (except for sedentary behaviour) at six months follow-up. Although there was no control group (without parents), the authors mentioned that one of the key component to success was the presence of the parent. Burke et al. [52] also reported positive outcomes by conducting a physical and nutrition program that included one parent. They concluded that parental involvement seemed to be very important to have an impact on long-term improvements. Wilson et al. [48] also reported the importance of parental involvement. They conducted a randomized intervention that promoted behaviour skills by dealing with communication, reciprocity of social support, group goal setting and behavioral competence. The intervention group consisted of low income and minority adolescents. Improvements on physical activity were measured during the intervention but could not be maintained at the two-week follow-up. The qualitative assessment (focus group) after the completion of their program showed that the following factors were impediments for physical activity: (1) competing demands at home; (2) not being motivated without teachers; (3) lack of parent involvement; and (4) environmental barriers, such as “bad weather” and “safety concerns” [48]. The failure or success of a study depends not only on the study design, but also on the social environment and background.

Another intriguing factor for intervention success we noted was gender differences. Horne et al. [43] reported that both boys and girls had short-term improvements in physical activity with their intervention; however, in the long term, only girls maintained their improvement. The intervention consisted of measuring daily steps with a pedometer, and feedback was provided to those that reached or outperformed their goals. The authors suggested that their findings might be partly explained by the hypothesis that girls could be more sensitive to reinforcers like self-monitoring, rewards and daily record procedures than boys would be. Contrary to Horne et al. [43], Manios et al. [37] reported that boys had a significant improvement in physical activity while the girls did not. The authors suggest that a possible explanation for these discrepancies is the way in which the physical activity classes were delivered: classes focused on non-competitive recreational activities that were created to promote self-mastery and efficacy with the joy of reaching the demands of exercising. It is therefore important that study designs respect the gender difference in creating physical activity programs.

## 5. Study Limitations

Comparisons across studies are challenging given the important heterogeneity across study designs, local contexts, geographic settings and social and cultural backgrounds of the samples. Some studies were restricted to overweight or obese participants, while others were not. Populations also varied substantially by socio-economic status. Hence, this should be considered when interpreting the results of this review. Furthermore, many environmental clinical approaches were not included, because they were aimed at a broad population, as opposed to only a pediatric population. Since only studies that targeted children exclusively were selected, the impact of environmental approaches on the pediatric population could have been misrepresented. The number of studies using an environmental approach relative to the two other categories was small, and thus, conclusions for this category may be less generalizable.

## 6. Conclusions

In conclusion, different categories of intervention, such as behavioral and social, informational or environmental approaches, were analyzed in this review. Although they are very different in their conception and application, they were generally successful in improving physical activity and cardiovascular risk factors. The main challenge in intervention implementation is not only to increase physical activity, but to maintain the beneficial effects once the intervention is completed and to ensure adherence. In order to achieve the best outcome, the intervention should take into account important factors, such as the length of the intervention or the frequency of follow-up. It should include psycho-social support, such as parental involvement, in order to achieve the desirable outcome.

## Figures and Tables

**Table 1:** Physical activity interventions with control groups (*n* = 31).

Type of INT	Duration (Frequency)	Sample (Characteristics)	FU	Articles and Description	Drop-Out (%)	Results Date	Outcomes and Results					
							Ant	PA	SB	Diet	Psy	Others
**Behavioral and Social Approach**	20 wk (3×/wk, 20 min)	97 (4.1 y)	6 wk	*Jones (2011)* [51] Structured lessons and unstructured activities with equipment	11	6 mo FU	↔	↑	↔			Movement skills: ↑
	9 mo	4997 (6.3 y)	4 y	*Plachta-Danielzik (2007) [26]* Messages to increase PA, healthy eating and decrease SB	65	4 y FU	↔	↔	↔			Incidence of OW: ↑
	9 mo	4997 (6.3 y)	8 y	*Plachta-Danielzik (2011)* [27] Messages to increase PA, healthy eating and decrease SB	76	8 y FU	↔	↔	↔			Lifestyle: ↔ BP: ↔
	4 mo (5×/wk, 40 min)	79 (7–11 y, OB)	4 mo	*Gutin (1999)* [50] Physical training 5 d/wk, 40 min	11	End of INT	↑	↑		↔		Insulin: ↑ Leptin: ↑ Lipids: ↔ BP: ↔ Left ventricle: ↔ Hemodynamic: ↔
						4 mo FU	↔	↑		↔		Insulin: ↔ Leptin: ↔ Lipids: ↔ BP: ↔ Left ventricle: ↔ Hemodynamic: ↔
	(12×)	386 (7–11 y)	14 wk	*Hardman (2011)* [44] (1) Peer-modeling, pedometer step goals and tangible rewards to increase PA; (2) same INT, without rewards	39	During INT	↔	(1) ↑↑ (2) ↑				
						14 wk FU	↔	(1) ↑ (2) ↑↑				
	12 wk (4×)	163 (7.4 ± 1.6 y)	9 and 15 mo	*McCallum (2007)* [21] LEAP: GP consultations targeting change in nutrition, PA, SB supported with materials	10	9 mo FU	↔	↑		↑	↔	
						15 mo FU	↔	↔		↑	↔	
	12 wk (4×)	258 (7.4 ± 1.6 y)	6 and 12 mo	*Wake (2009)* [46] LEAP 2: Consultations with GP targeting change in nutrition, PA, SB supported with materials	6	6 mo FU	↔	↔	↔	↔		
						12 mo FU	↔	↔	↔	↔		
	20 wk (5×/wk, 30 min)	238 (8–9 y)	12 mo	*Hopper (2005)* [53] School-based and home programs for PA and nutrition	N/A	End of INT	↔	↔		↑		Knowledge: ↑ Cholesterol: ↔
						12 mo FU	↔	↔		↑		Knowledge: ↔ Cholesterol: ↔
	20 wk (15×, 90 min)	70 (8 y)	6 mo	*Kalavainen (2007)* [57] Nutrition and PA education, decrease SB and behavioral therapy	3	End of INT	↑					
						6 mo FU	↔					
	(8×)	100 (9–11 y)	12 wk	*Horne (2009)* [43] Peer modeling, rewards and pedometer feedback to increase PA	11	During INT		♂: ↑ ♀: ↑				
						12 wk FU		♂: ↔ ♀: ↑				
	10 wk (7×, 40–120 min)	84 (9 y, OW and OB)	12 mo	*Toruner (2010)* [58] Games and skill building activities on perception of competence, PA, nutrition, SB and goal setting + parent training	4	3 mo FU	↔					Children knowledge: ↑ Parents knowledge: ↑
						12 mo FU	↑					
	24 wk (3×/wk, 90 min)	1119 (9.4 ± 0.7 y)	9 mo	*Martinez Vizcaino (2008)* [61] Afterschool recreational, non-competitive PA	7	9 mo FU	↔					Triceps skinfold: ↑ Apolipoprotein B: ♂: ↓, ♀: ↔ Apolipoprotein A: ♂: ↑, ♀: ↔ Cholesterol: ↔ Triglycerides: ↔ BP: ↔ except ♂: ↓dBP
	6 h, (1×)	153 (10–12 y)	2 mo	*Baranowski (2011)* [42] 2 games to provide practical knowledge related to change goals	13	2 mo FU	↔	↔		↑		
	6 mo (18×, 2 h)	116 (INT 10.3 ± 1.3 y)	6 mo	*Sacher (2010)* [24] Group education and PA session (MEND)	31	End of INT	↑	↑	↑		↑	Cardiovascular fitness: ↑
						6 mo FU	↑	↑	↔		↑	Cardiovascular fitness: ↑
	20 wk (4×/wk, 19 min )	800 (11 y)	6 mo	*Burke (1998)* [52] 2 INT: (1) Daily fitness sessions + nutrition; (2) Enriched daily fitness sessions for “at risk” children + nutrition	10	End of INT	(1) ♂: ↑ ♀: ↔	↔	↔	↔		Cardiovascular fitness: ↑ Cholesterol: ↑ BP: ↔ Triceps skinfold ♀: ↑, ♂: ↔
							(2) ♂: ↔ ♀ :↑	↔	↓	↔		Cardiovascular fitness: ↑ Cholesterol: ↑ for ♀ only BP: ↔Triceps skinfold: ↑
						6 mo FU	(1) ♂: ↔ ♀: ↔	↔	↔	↔		Cardiovascular fitness: ↑ for ♀ Cholesterol: ↑ BP: ↔ Triceps skinfold ♀: ↑, ♂: ↔
							(2) ♂: ↔ ♀ :↔	↔	↔	↔		Cardiovascular fitness: ↑ for ♀ Cholesterol: ↑ BP: ↔ Triceps skinfold: ↔
	17 wk (4×/wk, 2 h)	1422 (11.3 ± 0.6 years, low income and minority)	2 wk	*Wilson (2011)* [48] Promotion of behavioral skills to increase PA	25	Mid-INT		↑			↑	
						2 wk FU		↔			↑	
	4 wk (1×/wk, 40 min + 3× 90 min)	729 (12 y)	3 and 7 mo	*Cui (2012)* [19] Workshops and lessons focused on food choices, PA and SB	6	3 mo FU		↔	↔			
						7 mo FU		↔	↑			
	12 wk (1×/wk, 90 min)	291 (INT 12.2 ± 1.1 y, Portuguese)	3 and 9 mo	*Araujo-Soares (2009)* [22] Sessions on health behaviors, PA, diet, physical and psychosexual development, prevention of risk behaviors (STD, smoking, alcohol), assertiveness	33	End of INT		↔			↔	Coping planning: ↑
						3 mo FU		↑			↔	
						9 mo FU		↑↑			↔	
						5 mo FU		↔			↑	
							(2) ↑		↔			BP: ↑, mostly sBP Triglycerides: ↑ Insulin resistance and level: ↔ Glucose: ↔ Cholesterol: ↔
	6 d (1h, 12×)	1057 (12.4 ± 1.2 y)	3, 6 and 12 mo	*Jemmott (2011)* [18] Increase fruits and vegetables intake and PA, decrease smoking and alcohol	3	3 mo FU		↑↑↑		↑↑↑	↑↑↑	Knowledge: ↑↑↑ Drugs: ↔ Alcohol: ↔
						6 mo FU		↑↑		↑↑	↑↑	Knowledge: ↑↑ Drugs: ↔ Alcohol: ↔
						12 mo FU		↑		↑	↑	Knowledge: ↑ Drugs: ↔ Alcohol: ↔
	10 wk (8×, 15 min)	883 (INT 12.7 ± 0.7 y)	4 and 24 mo	*Ezendam (2012)* [45] Computer-tailored intervention with feedback	14	4 mo FU	↔	↓	↔	↑		
						24 mo FU	↔	↔	↔	↑		
	(3×)	1213 (12.7 ± 0.5 y)	1 and 6 mo	*Prins (2012)* [17] (1) Lessons + homework on PA self-regulation with feedback; (2) same INT without feedback	32	1 mo FU	↔	↔				
						6 mo FU	↔	↔				
	9 wk (1×/wk, 55 min)	473 (12.8 ± 1.1 y)	6 mo	*Latif (2011)* [20] 2 INT: (1) increase fruits and vegetables consumption; (2) increase PA, skill building activities for both and goal settings	24	End of INT		↑		↑↑FJ ↑LV	↔	
						6 mo FU		↑↑		↑FJ ↓LV	↔	
	60 min (1×)	320 (13.1 ± 0.8 y)	3 mo	*Haerens (2007)* [41] Computer tailored intervention to increase PA with feedback	12	3 mo FU		↑ School PA				but ↔ leisure and total PA
	20 min (2×)	1053 (14.5 ±1.4 y)	1 and 3 mo	*De Bourdeaudhuij (2010)* [16] Computer-tailored advice on PA	53	1 mo FU		↑				
						3 mo FU		↑↑				
	12 min (1×)	604 (15.3 ± 1.09 y)	3 and 12 mo	*Werch (2005)* [15] One on one screening and consultation on PA and alcohol use	15	3 mo FU		↑				Alcohol: ↑ Drug use: ↑ Cigarette: ↔
											
						12 mo FU		↔				Alcohol: ↑ Drug use: ↔ Cigarette: ↑
**Informational approach**	72 mo (2×/wk, 45 min + 13–17 h)	716 (start of INT: 6.3 ± 0.3 y)	48 mo	*Manios (2006)* [37] 4 y FU of a multi-component workbooks, nutrition and PA education and PA lessons (end of INT results and outcomes are cited in *Manios (2002)* [54])	41	End of INT	↔	↑		↓		Lipids: ↑ Cardiovascular fitness: ↑
					41	48 mo FU		♂: ↑ ♀: ↔				
	1 month	579 (INT 10.2 ± 1.0 y)	3 mo	*Francis (2010)* [33] Lessons on nutrition education and PA	18	3 mo FU		↔		↑		Knowledge: ↑
	5–7 days (2 h)	459 (12.5 ± 0.6 y)	3 mo	*Spruijt-Metz (2008)* [32] Theory-based classroom media to increase PA and decrease SB	N/A	3 mo FU	↔	↔	↑		↑	
	1 hour	693 (13.8 ± 1.4 y)	4 wk	*Schwarzer (2010)* [31] Theory-guided intervention to increase PA	23	4 wk FU		↑				
**Environmental approach**	36 mo	5106 (start of INT: 8–11 y)	5 y	*McKenzie (2003)* [39] CATCH 5 y FU: food service modification, increase PA and classroom health curricula (during INT results and outcomes are cited in *Webber (1996)* [40])	21	During INT (30 mo)	↓	↑	↑	↑		BP: ↔ Cardiovascular fitness: ↔ Skinfold: ↔ Lipids: ↔ Cholesterol: ↑
					
					N/A	5 y. FU		↑				
	24 mo	3502 (11–14 y, adolescent girls)	12 mo	*Webber (2008)* [56] TAAG: increase opportunities, support, incentives for PA	N/A	End on INT	↔	↔	↔			Cardiovascular fitness: ↔
					N/A	12 mo FU	↔	↑	↑			Cardiovascular fitness: ↔

Notes: Ant: anthropometric measures; BMI: body mass index; BP: blood pressure; d: days; dBP: diastolic blood pressure; FJ: fruit juice; FU: follow-up; HW: healthy weight; INT: intervention; LV: low fat vegetable; mo: months; N/A: not available; OB: obese; OW: overweight; PA: physical activity; Psy: psychological; SB: sedentary behaviour; sBP: systolic blood pressure; STD: sexually-transmitted disease; wk: weeks; y: years; ↔: no significant difference; ↑: significant increased outcomes, in terms of a *better* health status; ↓: significant decreased outcomes, in terms of a *worse* health status. LEAP: live, eat and play; CATCH: Child and Adolescent Trial for Cardiovascular Health; TAAG: Trial of Activity for Adolescent Girls.

**Table 2:** Physical activity interventions without control groups (*n* = 19).

Type of INT	Duration (Frequency)	Sample (Characteristics)	FU	Articles and Description	Drop-Out (%)	Results Date	Outcomes and Results					
							Ant	PA	SB	Diet	Psy	Others
**Behavioral and social approach**	4 mo (5×/wk, 5–10 min)	213 (5–11 y)	3 mo	*Erwin (2011)* [49] Teacher-directed classroom-based PA breaks	50	End of INT		↑				
						3 mo FU		↑				
	N/A	43 (8–14 y)	N/A	*Hermann (2006)* [29] Afterschool education and gardening on nutrition and PA	N/A	N/A		↑		↑		
	12 wk (1×/wk, 90 min)	1529 (8–11 y, girls)	5 mo	*Pettee Gabriel (2011)* [23] Training for running event and physical, psychological and social development	43	End of INT		↑			↑	
						5 mo FU		↔			↑	
	23 wk (10×, 2 h)	165 (8 y, OW and OB)	6 and 12 mo	*Okely (2010)* [60] (1) Parent-centered diet program; (2) child-centered PA program; or (3) combination of both; all include face-to-face sessions, homework and a prevention program	36	6 mo FU	↑					Lipids: ↔ Insulin: ↑ for group (1) only Glucose: ↔ BP: ↔
						12 mo FU	↑					Lipids: ↔ Insulin: ↔ Glucose: ↔ BP: ↑sBP from group (2) only
	6 mo (20 meetings, 1 h)	63 (9.8 ± 1.3 y OB)	6 mo	*Epstein (2004)* [25] Stimulus control or reinforcement to reduce SB and substitute for PA, enhance light diet and behavior change techniques	3	End of INT	↑	↑	↑	↑		
						6 mo FU	↑	↔	↔	↔		
	12 wk (5×/wk, 10–30 min)	319 (10 y)	6 mo	*Balas-Nakash (2010)* [59] 2 INT: (1) 10 min of aerobic exercises; (2) 30 min of aerobic exercises	33	6 mo FU	(1) ↑		↔			BP: ↑, mostly dBP → Triglycerides: ↑ Insulin resistance and level: ↔ Glucose: ↔ Cholesterol: ↔
							(2) ↑		↔			BP: ↑, mostly sBP Triglycerides: ↑ Insulin resistance and level: ↔ Glucose: ↔ Cholesterol: ↔
	9 mo (6×/wk, 30 min)	140 (INT 10.1 y)	3 wk	*DeVault (2009)* [62] PA (aerobic, strength and endurance) and nutrition lessons	39	3 wk FU	↔			↑	↑	Knowledge: ↑ Aerobic fitness: ↑
	12 wk (3×/wk)	30 (12.6 ± 1.0 y, Hispanic and African American girls)	1 wk	*Colchico (2000)* [47] Activities and exercise sessions	0	1 wk FU	↑	↑		↑		Cardiovascular fitness: ↑ Muscle strength: ↑ Flexibility: ↑
	10–25 min (1×)	454 (13–14 y)	3 mo	*Werch (2003)* [14] Prevent alcohol use and promote PA	3	3 mo FU		↑			↑	Alcohol: ↑
	10 mo (8 h/wk)	30 (13.5 ± 2.1 y, OB)	6 mo	*Deforche (2004)* [28] Dietary, PA, psychological support, medical supervision	20	During INT	↑	↑	↑		↔	Social support: ↑
						6 mo FU	↑	↓	↓		↔	Modelling for siblings: ↓ Modelling for parents: ↔ Social support: ↓
**Informational approach**	48 mo	2215 (8–12 y)	9 mo	*McDermott (2010)* [36] Media campaign to encourage PA	N/A	9 mo FU		↑				
	1×	509 (INT 8.3 y)	Several wk	*Stahl (2011)* [30] One motivational clinical visit to increase PA, healthy eating and decrease SB	25	Several wk FU		↑	↑	↑		
	11 mo	57 (8.8 ± 0.8 y, Chinese American)	2 wk, 1 and 6 mo	*Chen (2008)* [35] Mailed education package and tailored materials	26	6 mo FU	↑	↑	↓	↑		Child Knowledge: ↑ Parent Knowledge: ↔
	3 mo	295 (9–13 y)	3 mo	*Balamurugan (2005)* [34] Paid radio advertisement to promote PA	N/A	3 mo FU		↑			↑	
**Environmental approach**	24 mo	554 (start of INT: 7.7 ± 1.8 y)	24 mo	*Taylor (2008)* [63] 2 y FU of APPLE: encouraging healthy eating and increase PA with community activity coordinators (end of INT results and outcomes are cited in *Taylor (2007)* [55])	24	During INT	↑					sBP: ↑
						End of INT	↑	↑	↔	↑		BP: ↔ Cardiovascular fitness: ↔
						24 mo FU	↑			↑		
	12 mo	1594 (17.7 ± 0.6 y, adolescent girls)	36 mo	*Pate (2007)* [38] LEAP 2: increase PA through changes in practices and school environment	34	36 mo FU	↔	↑				

Notes: Ant: anthropometric measures; APPLE: A Pilot Programme for Lifestyle and Exercise; BMI: body mass index; BP: blood pressure; CATCH: Child and Adolescent Trial for Cardiovascular Health; d: days; dBP: diastolic blood pressure; FJ: fruit juice; FU: follow-up; HW: healthy weight; INT: intervention; LEAP: live, eat and play; LV: low fat vegetable; mo: months; N/A: not available; OB: obese; OW: overweight; PA: physical activity; Psy: psychological; SB: sedentary behaviour; sBP: systolic blood pressure; STD: sexually-transmitted disease; TAAG: Trial of Activity for Adolescent Girls; wk: weeks; y: years; ↔: no significant difference; ↑: significant increased outcomes, in terms of a *better* health status; ↓: significant decreased outcomes, in terms of a *worse* health status.
